# Efficacy of Preventive Pressurized Intraperitoneal Aerosol Chemotherapy in Patients With Locally Advanced Gastric Cancer: Protocol for a Prospective Controlled Trial

**DOI:** 10.2196/78053

**Published:** 2025-11-11

**Authors:** Altay Kerimkulov, Talgat Uskenbayev, Tomiris Sarina, Meiram Mamlin, Sanzhar Shalekenov, Gulfairus Yessenbayeva, Temirlan Kainazarov, Zhandos Burkitbayev, Baiduisen Ussipbekov, Dina Kovalchuk, Akhror Rakhmankulov, Aidana Rakhmankulova, Abduzhappar Gaipov

**Affiliations:** 1 National Research Oncology Center Astana, null Kazakhstan; 2 Municipal hospital No. 1 Astana, null Kazakhstan; 3 Nazarbayev University Astana, null Kazakhstan

**Keywords:** gastric cancer, peritoneal metastasis, pressurized intraperitoneal aerosol chemotherapy, PIPAC, chemotherapy, HIPEC, hyperthermic intraperitoneal chemotherapy, oncology, intraperitoneal treatment

## Abstract

**Background:**

Gastric cancer is a serious health issue both globally and in Kazakhstan. Worldwide, gastric cancer ranks fifth in incidence, with the highest rates reported in East Asia, the Andes of South America, and Eastern Europe, while the lowest rates are reported in North America, Northern Europe, Africa, and Southeast Asia. Over 70% of the cases occur in resource-limited countries. In Kazakhstan, gastric cancer ranks third in morbidity and second in mortality, representing a serious clinical and social problem. Annually, 1600 deaths from malignant gastric neoplasms are registered. Peritoneal metastasis is considered one of the most severe complications of gastric cancer and has long been regarded as its terminal stage. The average life expectancy of patients with peritoneal metastasis is only 3-6 months, reflecting a high mortality rate and the limited efficacy of current treatments.

**Objective:**

We hypothesize that the prophylactic use of pressurized intraperitoneal aerosol chemotherapy (PIPAC) may reduce the incidence of peritoneal metastasis. The aim of this study is to evaluate the incidence of peritoneal metastasis following prophylactic PIPAC in patients with gastric cancer. The primary goal of this research is to evaluate whether adding prophylactic PIPAC to standard treatment before neoadjuvant chemotherapy can reduce the incidence of peritoneal metastasis in patients with locally advanced gastric cancer.

**Methods:**

This study is a single-center nonrandomized controlled trial with a planned enrollment of 160 patients. All participants will be included in one of the 2 groups: intervention group (PIPAC + perioperative chemotherapy + gastrectomy with D2 lymphadenectomy) or control group (perioperative chemotherapy + gastrectomy with D2 lymphadenectomy). The primary end point is the incidence of peritoneal metastasis, and the secondary end points are overall survival, recurrence-free survival, treatment-related adverse events, and personal satisfaction.

**Results:**

As of September 2025, this study is funded and recruiting; 102 participants have already been enrolled. Recruitment is planned to be performed between January 2025 and December 2026. No interim analyses have been performed; primary results are planned for Q1 2027. The manuscript is expected to be published by Q4 2027.

**Conclusions:**

Compared to standard chemotherapy, PIPAC has been reported to significantly improve survival rates in patients with peritoneal metastasis of gastric origin. We suggest that the neoadjuvant use of PIPAC may reduce the incidence of peritoneal metastasis and improve long-term survival outcomes. The results of our study will provide key information on the practicality and viability of PIPAC as a prophylactic technique for preventing the progression of gastric cancer.

**Trial Registration:**

ClinicalTrials.gov NCT06784765; https://clinicaltrials.gov/study/NCT06784765

**International Registered Report Identifier (IRRID):**

PRR1-10.2196/78053

## Introduction

Gastric cancer poses a serious health issue both globally and in Kazakhstan [1,2]. Worldwide, it ranks fifth in incidence, with the highest rates seen in East Asia, the Andes of South America, and Eastern Europe, while the lowest are reported in North America, Northern Europe, Africa, and Southeast Asia [1,3,4]. Over 70% of the cases occur in resource-limited countries. In Kazakhstan, gastric cancer ranks third in incidence and second in mortality among all malignancies, causing approximately 1600 deaths annually, highlighting its significant social and clinical burden [1,2,4]. One of the most serious complications of gastric cancer is peritoneal dissemination, which has long been considered a terminal stage of the disease, resistant to curative treatment [5,6]. Peritoneal metastasis (PM) develops in a considerable proportion of patients, even among those receiving multimodal therapy, and poses a major challenge in oncology. The median overall survival for patients with PM is only 3-6 months, underscoring the urgent need for more effective therapeutic approaches [5]. PM results from tumor cell detachment, survival in the peritoneal cavity via anoikis resistance, adhesion to mesothelial surfaces, and invasion into submesothelial tissues. Hypoxic environments such as milky spots and molecular pathways, including integrins and CXCR4, facilitate colonization [7]. Systemic chemotherapy remains the cornerstone of treatment for gastric cancer with PM. However, its efficacy is limited, particularly against peritoneal lesions, due to poor drug penetration into the peritoneal tissues [8,9].

In response to these limitations, new strategies such as the combination of cytoreductive surgery and hyperthermic intraperitoneal chemotherapy (HIPEC) have been developed [10]. Intraperitoneal drug administration allows for higher local drug concentrations while reducing systemic toxicity, potentially improving clinical outcomes [11,12]. Despite its promise, HIPEC has notable limitations in the context of gastric cancer. Its application is technically challenging, associated with significant morbidity, and its long-term benefits remain under debate. These drawbacks highlight the need for alternative, less invasive, and more effective intraperitoneal treatment modalities.

Pressurized intraperitoneal aerosol chemotherapy (PIPAC), proposed by Reymond et al [13] in 2011 represents an innovative alternative. PIPAC delivers chemotherapeutic agents in aerosol form under high pressure into the peritoneal cavity, enhancing drug penetration into the peritoneal tissues and improving drug distribution. This method combines the advantages of effective local delivery with reduced systemic toxicity [13,14]. According to Alyami et al [15], PIPAC improves drug penetration into the peritoneum and minimizes systemic exposure, offering a promising alternative to systemic chemotherapy [15]. Several studies have demonstrated the safety and efficacy of PIPAC for various types of PM [16]. Another study included 25 patients who were available for analysis of the primary end point [17]. Of these, 10 (40%) patients showed a radiological complete or partial response or stable disease. The median overall survival by intention to treat was 6.7 months, and the median time to progression was 2.7 months. Complete or major regression on histology was observed in 9 (36%) out of 25 patients (intention to treat) or in 6 out of 6 patients (100% per protocol) in the group with full protocol adherence. There were no unexpected serious adverse effects, no treatment-related deaths, no grade 4 toxicity by the common terminology criteria for adverse events, and only 3 (12%) patients experienced grade 3 toxicity. Changes in QLQ-C30 scores during PIPAC C/D therapy were minor and not statistically significant. Studies show that compared to HIPEC, PIPAC offers advantages such as minimal invasiveness, repeatability, reduced toxicity, and improved drug absorption.

According to Ramalho-Vasconcelos et al [18], patients with gastric cancer who had undergone 3 or more PIPAC procedures had a shorter hospitalization period (mean difference = –1.2) and significantly improved survival (mean difference = 6.0) [18]. Aksenov et al [19] performed 20 cytoreductive surgeries, wherein intraperitoneal aerosol chemotherapy was combined with limited PM, and they obtained the following results: 3 patients showed partial response, 17 showed stabilization of the tumor process at the primary site after preoperative treatment, and 50% of the patients had a complete response in the peritoneal tumor sites [19]. The median overall survival was 27 months, the median recurrence-free survival was 7 months, and the overall 1-year and 2-year survival rates were 95% and 60%, respectively. Luksta et al [20] reported that the postoperative complication rate was less than 5% with no complications threatening life.

By aerosolizing chemotherapy within the peritoneal cavity, PIPAC achieves more even distribution into anatomic recesses and enhances tissue penetration with sustained local exposure. It also limits systemic absorption, thereby reducing toxicity, and can be safely repeated, allowing consistent evaluation of treatment response [21]. Moreover, Solass et al [14] showed that with the treatment of doxorubicin, PIPAC showed no significant adhesions and was appropriate to be repeated 6x, 4x, and 2x [14]. Another study applied PIPAC in combination with cisplatin (7.5 mg/m^2^) and doxorubicin (1.5 mg/m^2^) every 6 weeks with no adverse reactions, no treatment-related deaths, and no grade 3 and grade 4 toxicities [17].

Nevertheless, the role of PIPAC in gastric cancer treatment, particularly in preventing peritoneal dissemination, requires further clinical evaluation. We hypothesize that the prophylactic use of PIPAC may reduce the incidence of PM. The aim of this study is to evaluate the incidence of PM following prophylactic PIPAC in patients with gastric cancer.

PIPAC is hypothesized to enhance chemotherapy penetration, improving local disease control, and potentially reduce the incidence of PM compared to standard treatment alone. The primary aim of this study is to assess the efficacy and safety of PIPAC in the preoperative management of patients with locally advanced gastric cancer without PM in combination with conventional treatment. A comparative analysis between the experimental and control groups will be conducted to identify the potential benefits and assess the effectiveness of this novel therapeutic approach.

## Methods

### Study Design

This study is designed as a single-center, nonrandomized controlled trial aimed at evaluating the efficacy and safety of PIPAC as a preventive strategy in the management of locally advanced gastric cancer. In line with IDEAL stage 2b (exploration), we use a prospective, nonrandomized controlled cohort to (1) standardize the procedure and perioperative workflow, (2) refine outcome definitions (especially peritoneal-metastasis ascertainment), and (3) estimate the effect sizes and variability to inform a later multicenter randomized controlled trial. The study will be conducted at the National Research Oncology Center, Kazakhstan, enrolling a total of 160 patients. Participants will be parallelly assigned to either the PIPAC intervention group or the control group in a 1:1 ratio. Patients receive PIPAC + standard care versus standard care when they consent to prophylactic laparoscopy-integrated PIPAC after shared decision-making. Personal information will not be available for the public and will be encrypted into special codes.

Since a single-centered nonrandomized approach can lead to several limitations, the adjustment for confounding will be done. First, we will minimize selection bias by consecutive screening, predefined allocation rules, and a log documenting the reasons for allocation. Second, we will adjust analytically by using propensity-score weighting to create comparable groups on pretreatment characteristics (age, sex, BMI, Eastern Cooperative Oncology Group score, clinical T/clinical N stage, tumor location/grade/Lauren type/linitis features, prior abdominal surgery, diabetes/Charlson comorbidity index, and perioperative timing). Outcomes will then be analyzed with robust standard errors, and PM will be adjudicated blinded to treatment. Baseline missing data will be handled with multiple imputation, and we will report preadjustment and postadjustment balance so that readers can verify that confounding was adequately controlled. This study is structured to ensure comprehensive data collection and analysis, enabling an in-depth understanding of PIPAC’s impact on disease progression, survival outcomes, and overall patient well-being. Given its nonrandomized design, rigorous inclusion and exclusion criteria have been established to minimize bias and ensure homogeneous study groups.

This protocol adheres to the SPIRIT (Standard Protocol Items: Recommendations for Interventional Trials) statement (Multimedia Appendix 1). Intervention reporting follows TIDieR (Template for Intervention Description and Replication; Multimedia Appendix 2). Given the nonrandomized controlled design, the TREND (Transparent Reporting of Evaluations with Nonrandomized Designs) checklist is provided (Multimedia Appendix 3).

### Study Population

To maintain a well-defined patient cohort, this study includes individuals who meet the inclusion and exclusion criteria. These eligibility criteria are designed to select a homogeneous study population that allows for a precise evaluation of PIPAC’s preventive potential while minimizing the confounding variables (Textbox 1).

**Inclusion and exclusion criteria for the study population.**

**Inclusion criteria**

- Age: 18-70 years in alliance with other studies in this area of research topic
- Diagnosis: Histologically confirmed locally advanced gastric adenocarcinoma (T3-4N0-3M0 gastric adenocarcinoma, according to TNM classification, confirmed by computed tomography, magnetic resonance imaging, or positron emission tomography-computed tomography)
- Performance status: Eastern Cooperative Oncology Group score of 0-2, indicating good to moderate functional status
- Peritoneal cytology: negative results for malignant cells from diagnostic laparoscopy, ensuring no evidence of peritoneal dissemination at baseline
- Staging laparoscopy

**Exclusion criteria**

- Distant metastases detected via imaging or cytological analysis
- Previous treatment with chemotherapy, radiotherapy, or surgical interventions for gastric cancer
- Severe comorbidities that contraindicate surgical procedures or systemic chemotherapy (eg, severe cardiovascular, hepatic, or renal dysfunctions; poorly controlled diabetes)
- Technical/anatomical issues: factors precluding safe laparoscopy/pressurized intraperitoneal chemotherapy (eg, dense adhesions after major abdominal surgery; clinically significant ascites not correctable)
- Hypersensitivity to cisplatin/doxorubicin
- Active infection: systemic infection or abdominal wall/intraperitoneal infection

### Allocation

At staging laparoscopy (time zero), all eligible patients are counseled using a standardized script and decision aid. Patients are allocated in the PIPAC + standard care if patient consents and (1) inclusion/exclusion remain met, (2) no day of contraindication, (3) a credentialed surgeon/device is available within 14 days. If any of the above conditions are not met or the patient declines, standard care is applied. We will keep a prospective allocation-reason log (refusal, clinical, capacity/scheduling, other). Crossovers remain in their intention-to-treat arm; a per-protocol analysis will be reported as sensitivity. All other care pathways are identical between the groups.

### Interventions

#### Intervention Group

Patients in the intervention group will undergo preventive PIPAC with cisplatin (10 mg/m²) and doxorubicin (2.1 mg/m²) prior to the initiation of neoadjuvant chemotherapy. It was chosen based on the treatment protocol of the Republic of Kazakhstan as well as the existing justifications of efficiency in other studies performed [22]. Standard perioperative chemotherapy is administered according to international guidelines using the FLOT (5-fluorouracil, leucovorin, oxaliplatin, and docetaxel) regimen. Surgical intervention includes curative-intent gastrectomy with D2 lymphadenectomy, performed after the completion of neoadjuvant therapy and patient reassessment.

#### Control Group

Patients in the control group will receive standard perioperative chemotherapy with the FLOT regimen, followed by gastrectomy with D2 lymphadenectomy, according to the same protocol as in the experimental group.

### Primary End Point

As a primary end point, we will control for the incidence of PM at 12 months and 24 months postsurgery. Computed tomography is scheduled for every 3 months for 2 years. Unless computed tomography data show clear signs of carcinomatosis, PM is confirmed via laparoscopy at month 12 and month 24 with histological assessment. Laparoscopy may be performed earlier if the imaging of the symptoms suggests PM. All PM events are centrally adjudicated, blinded to treatment (2 reviewers; third resolves discrepancies).

### Secondary End Points

**Figure 1:**
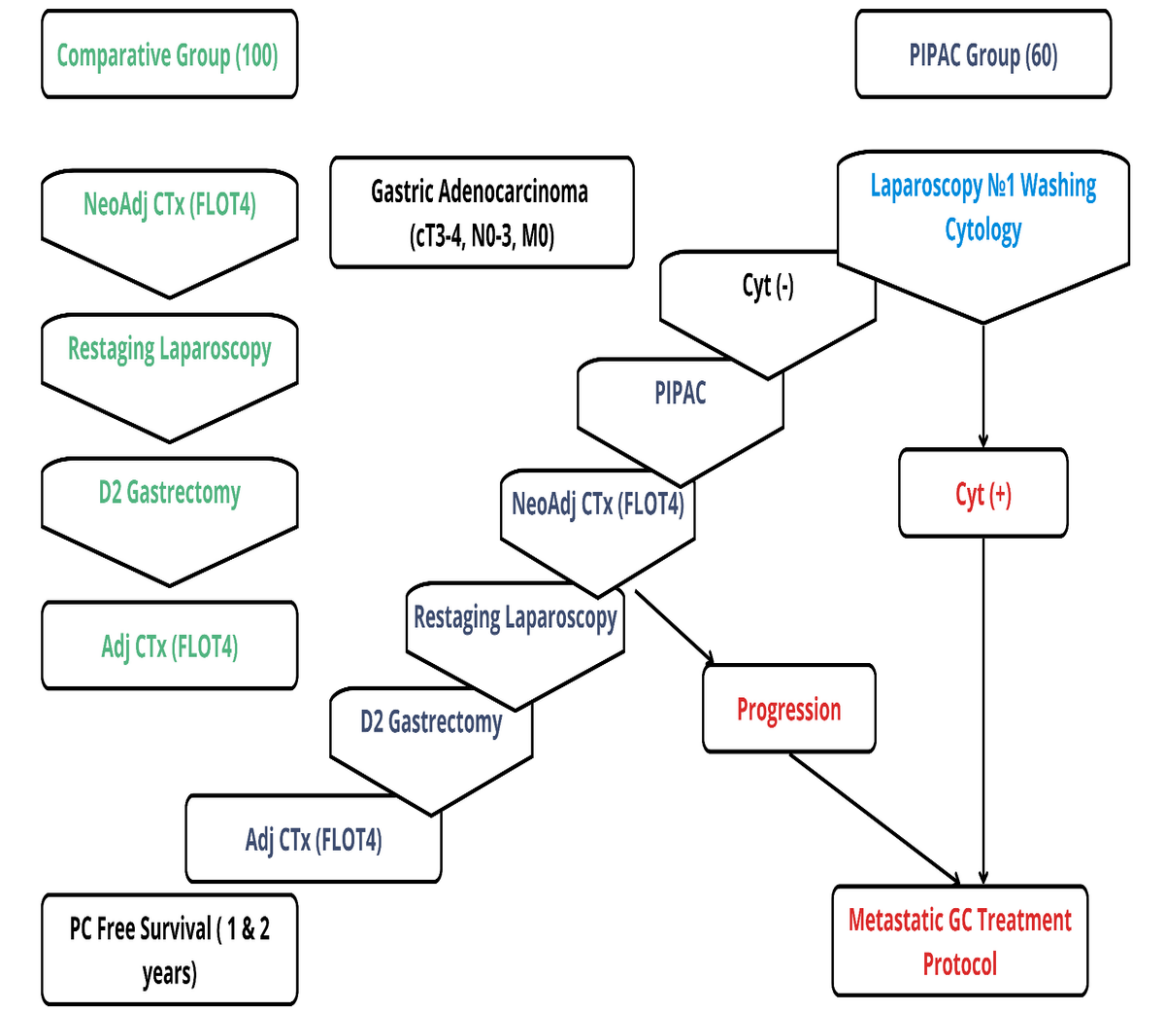
Study design. AdjCTx: adjuvant chemotherapy; cyt: cytology; FLOT: fluorocil, leucovorin, oxaliplatin, docetaxel; GC: gastric cancer; NeoAdjCTx: neoadjuvant chemotherapy; PC: peritoneal carcinomatosis; PIPAC: pressurized intraperitoneal aerosol chemotherapy.

There are several secondary end points to measure: (1) overall survival (time from time-zero to death from any cause) at 1, 3, and 5 years postsurgery; and (2) recurrence-free survival (time from time zero to first recurrence [any site, including peritoneal] or death, whichever occurs first) assessed at multiple time points. To assess overall survival and recurrence-free survival, patients will undergo endoscopic and radiological assessment every 3 months for 2 years. To control for the safety of the trial, the frequency of chemotherapy-related adverse events is graded according to the common terminology criteria for adverse events (version 5.0) and by Clavien-Dindo classification system for surgical complications after each course of chemotherapy. Grading will occur 30 days post-PIPAC, 90 days after gastrectomy, and throughout chemotherapy. Another type of assessment is the quality of life (QoL) assessment, using the standardized EORTC QLQ-C30 questionnaire, designed to measure various functional and symptom domains affecting patients with cancer. QoL will be assessed after the PIPAC procedure and radical surgery: baseline, presurgery, end of adjuvant therapy, and months 12 and 24. These end points will provide insights into both the clinical efficacy and safety profile of PIPAC, allowing for an evidence-based assessment of its role in gastric cancer management. Figure 1 shows the study scheme: staging laparoscopy with cytology, prophylactic PIPAC for cytology-negative patients, neoadjuvant FLOT×4, restaging laparoscopy, D2 gastrectomy, and adjuvant FLOT×4. Cytology-positive or progressing patients exit to metastatic care pathways. The primary end point is PM incidence at 12 or 24 months.

### Statistical Analysis

To ensure the robustness of the study findings, the following statistical methods will be applied at the analysis stage.

For sample size calculation, powering was based on the primary end point (death as a competing risk; primary analysis via Fine-Gray), while secondary end points (overall survival, recurrence-free survival, QoL) are exploratory. The log-rank/Schoenfeld approximation in MedCalc with 2-sided α=.05, N=160 (1:1) provided ~79.7% power to detect a 25%-30% reduction in PM incidence. To mitigate low power, we will focus on the estimation analysis by calculating risk difference/ratio with 95% CI, avoiding dichotomous claims.

For the primary analysis (overall survival and recurrence-free survival), the Kaplan-Meier method with Inverse Probability of Treatment Weighting -adjusted Cox/Fine-Gray with robust SEs will be used. Once it is done, log-rank test will be applied for comparing the survival curves between the intervention and control groups. Fisher exact test and Pearson chi-square test will be employed for categorical data analysis, ensuring statistical rigor in comparing treatment outcomes. Multiplicity for QoL is controlled via a false discovery rate (q=0.10), while the global health status at month 12 is prespecified as the primary QoL end point. Shapiro-Wilk test will be used to assess the normality of the data distributions, and Student 2-sided *t* test will be applied to analyze continuous variables with normal distribution, while the Mann-Whitney *U* test will be used for nonnormally distributed data. Data will be censored at the last follow-up visit, with additional sensitivity analyses conducted if necessary for the better handling of missing data. These analytical methods will allow for an objective evaluation of PIPAC’s effectiveness while ensuring statistical validity and the reproducibility of results.

### Ethical Considerations

This study was approved by the National Research Oncology Center Board (approval 051; April 24, 2024). Written informed consent will be obtained from all the participants. Participants have the right not to participate in the study and be treated by standard therapy protocol. Participant data will be deidentified before analysis. Each participant will be assigned a unique study ID; the linkage file connecting IDs to personal identifiers will be stored separately on an encrypted, access-restricted server and will be accessible only to authorized study personnel. No personal identifiers will be visible during data processing or analysis. For this study, participants receive no monetary compensation.

### Data and Safety Monitoring Board

An independent data and safety monitoring board (surgical oncologist, medical oncologist, biostatistician; with no study roles) oversees participant safety. The board works under a written charter (membership, conflicts, data access, decision rules). The board member receives unblinded safety reports from an independent statistician and meets quarterly/ad hoc to issue continue/pause/modify/stop recommendations to the sponsor/institutional review board. All additional information are available by request.

## Results

As of September 2025, this study is funded and recruiting; 102 participants have already been enrolled. Recruitment is performed between January 2025 and December 2026. No interim analyses have been performed; primary results are planned for Q1 2027. Data collection is ongoing; database lock/analysis is expected to occur in Q1-Q2 2027; and the manuscript is expected to be published by Q4 2027.

## Discussion

### Principal Findings

The use of PIPAC as a preventive method represents a promising strategy to reduce the incidence of PM in patients with locally advanced gastric cancer and to improve overall survival [23,24]. This study aims to evaluate the effectiveness of PIPAC in combination with standard perioperative therapy and to determine its potential advantages over currently employed treatment modalities. Current data indicate a high incidence of PM in gastric cancer, which significantly worsens prognosis and often renders radical treatment unfeasible. Standard approaches, including systemic chemotherapy and HIPEC, demonstrate limited efficacy, particularly against microscopic peritoneal implants. Unlike HIPEC, which requires intraoperative administration and is associated with considerable toxicity, PIPAC is a minimally invasive procedure that enables repeated delivery of chemotherapeutic agents into the peritoneal cavity with high bioavailability [15,25].

The pathophysiology of peritoneal carcinomatosis underscores the need for early, localized intervention. Tumor cell adhesion, anoikis resistance, and colonization of hypoxic niches such as milky spots occur before macroscopic lesions develop. By targeting this early dissemination phase, PIPAC may interrupt key metastatic steps and reduce peritoneal spread in high-risk patients [7]. Although PIPAC has already demonstrated promising results in the treatment of PM from various solid tumors, its role as a preventive approach remains insufficiently studied. This research emphasizes the early application of PIPAC in patients at high risk of peritoneal dissemination, which may reduce the likelihood of PM development in the postoperative period. If the hypothesis is confirmed, the findings could support the integration of PIPAC into standard treatment protocols for locally advanced gastric cancer and potentially reshape existing clinical practice [15].

However, several questions require further investigation. First, it is essential to determine the optimal PIPAC regimen, including the number of cycles, choice of chemotherapeutic agents, and their dosages. Second, the potential complications associated with repeated aerosol chemotherapy administration remain unclear. Third, it is crucial to assess the impact of PIPAC on patients’ QoL, particularly over the long term. Comparing the results of this study with data on HIPEC and systemic chemotherapy will enable a comprehensive evaluation of PIPAC’s effectiveness and help define its role within the multimodal treatment paradigm for gastric cancer. Additionally, the development of a novel device for aerosol drug delivery may enhance dosing accuracy, improve drug distribution within the peritoneal cavity, and potentially increase therapeutic efficacy [15,26].

Thus, this study holds significant clinical importance, as its findings could contribute to refining current strategies for the treatment and prevention of PM. If successful, it will serve as a foundation for future multicenter trials aimed at validating the results and further optimizing the method. These aspects underscore the relevance of the proposed research and its potential to improve oncological care for patients with locally advanced gastric cancer.

### Limitation and Potential Biases

Prophylactic PIPAC in cytology-negative potentially curable patients has an uncertain risk benefit: possible harms include laparoscopic complications, adhesions, port-site issues, and cumulative intraperitoneal chemotherapy toxicity. Any reduction in early peritoneal relapse may not translate into overall-survival benefit. As a single-center, nonrandomized study, selection/confounding, learning-curve effects, and PM ascertainment error remain possible despite standardized imaging and scheduled laparoscopy. We mitigate these via consecutive screening with a prospectively maintained allocation-reason log, standardized pathways and follow-up, propensity-score weighting with prespecified balance targets, blinded PM adjudication, and multiple imputation for baseline covariates. Analyses are estimation-focused (effect sizes with 95% CIs), and findings will inform a subsequent multicenter randomized controlled trial.

## Data Availability

The datasets generated or analyzed during this study are available from the corresponding author on reasonable request.

## Appendix

Multimedia Appendix 1 SPIRIT checklist.

Multimedia Appendix 2 TIDieR checklist.

Multimedia Appendix 3 TREND statement checklist.

Multimedia Appendix 4 ChatGPT conversations.

## Abbreviations

FLOT: 5-fluorouracil, leucovorin, oxaliplatin, and docetaxel
HIPEC: hyperthermic intraperitoneal chemotherapy
PIPAC: pressurized intraperitoneal aerosol chemotherapy
PM: peritoneal metastasis
QoL: quality of life
SPIRIT: Standard Protocol Items: Recommendations for Interventional Trials
TIDieR: Template for Intervention Description and Replication
TREND: Transparent Reporting of Evaluations with Nonrandomized Designs
