# Supplementary material for: Efficacy of Preventive Pressurized Intraperitoneal Aerosol Chemotherapy in Patients With Locally Advanced Gastric Cancer: Protocol for a Prospective Controlled Trial
Source: JMIR Res Protoc. 2025 Nov 11;14:e78053. doi: 10.2196/78053 (PMC12648122; doi:10.2196/78053)
Supplement: Multimedia Appendix 4 [file resprot_v14i1e78053_app4.docx]

PIPAC prophylactically in non-metastatic gastric cancer| find related scientific articles studies on this topic

**ChatGPT said:**

I searched for studies on *prophylactic / preventive PIPAC in non-metastatic gastric cancer* (or settings close to that). I didn’t find many completed trials, but there are a few ongoing trials, retrospective data, and safety studies. Here’s a summary of what *is* known, what’s in progress, and gaps — useful for your protocol justification or literature review.

**What existing literature says**

| **Study / Type** | **Setting** | **Key findings / relevance** | **Limitations regarding prophylactic PIPAC in non-metastatic GC** |
| --- | --- | --- | --- |
| **“Early postoperative outcomes of staging laparoscopy …”** (Teixeira Farinha et al., 2022) [BioMed Central](https://bmcsurg.biomedcentral.com/articles/10.1186/s12893-022-01572-5?utm_source=chatgpt.com) | Retrospective comparative: staging laparoscopy *with* PIPAC vs laparoscopy alone, in patients with high-risk oesogastric cancer (GOJ, gastric) undergoing staging laparoscopy. [BioMed Central](https://bmcsurg.biomedcentral.com/articles/10.1186/s12893-022-01572-5?utm_source=chatgpt.com) | PIPAC added to laparoscopy was safe and tolerated similarly to laparoscopy alone. No increase in early postoperative morbidity. Suggests feasibility in a neoadjuvant / prophylactic-like setting. [BioMed Central](https://bmcsurg.biomedcentral.com/articles/10.1186/s12893-022-01572-5?utm_source=chatgpt.com) | Does not report long-term outcomes (relapse, peritoneal metastasis rate), unclear effect on disease-free survival. Also, this isn’t a prospective randomized design. Some patients had suspected peritoneal disease; not strictly “no peritoneal disease” prophylactic use. |
| **Lithuanian PIPAC program, GC with peritoneal metastases** (Luksta et al., 2024) [mdpi.com](https://www.mdpi.com/2072-6694/16/17/2992?utm_source=chatgpt.com) | Retrospective cohort of patients with *gastric cancer with peritoneal metastasis (PM)*, treated with PIPAC (repeated). [mdpi.com](https://www.mdpi.com/2072-6694/16/17/2992?utm_source=chatgpt.com) | Showed PIPAC is feasible and safe; some decreases in peritoneal carcinomatosis index (PCI) and ascites although not statistically significant. Median survival after PM ~12.5 months. [mdpi.com](https://www.mdpi.com/2072-6694/16/17/2992?utm_source=chatgpt.com) | Not preventive — patients already have PM. So doesn’t directly inform prophylactic use. Also retrospective, small numbers. |
| **Systematic review/meta-analysis** (PIPAC & GC PM) — R. Chen et al., 2025 [pmc.ncbi.nlm.nih.gov](https://pmc.ncbi.nlm.nih.gov/articles/PMC12167634/?utm_source=chatgpt.com) | Review of ~18 articles of PIPAC use in gastric cancer with PM | Summarizes safety, tolerability, some signals of efficacy; confirms gaps in high-quality evidence and that most data are in palliative / metastatic settings. [pmc.ncbi.nlm.nih.gov](https://pmc.ncbi.nlm.nih.gov/articles/PMC12167634/?utm_source=chatgpt.com) | Again, not about prophylaxis in cancers without existing peritoneal spread. |
| **PIPAC for gastrointestinal malignancies** (SK Daniel et al., 2023) [pmc.ncbi.nlm.nih.gov](https://pmc.ncbi.nlm.nih.gov/articles/PMC10650315/?utm_source=chatgpt.com) | Review paper discussing PIPAC in GI cancers including gastric | Provides background on typical dosages, how PIPAC is administered, what the current practice is, etc. Useful for methodologic justification. [pmc.ncbi.nlm.nih.gov](https://pmc.ncbi.nlm.nih.gov/articles/PMC10650315/?utm_source=chatgpt.com) | Also largely in metastatic or peritoneal carcinomatosis settings rather than prevention of peritoneal metastasis. |

**Ongoing / Planned Clinical Trials**

These are trials or protocols that are relevant because they *are* looking at adding PIPAC earlier in the disease, possibly for prevention or reduced metastatic risk.

| **Trial / Registration** | **Objective / Hypothesis** | **Design / Status** | **How it relates to prophylactic use** |
| --- | --- | --- | --- |
| **Preventive Use of PIPAC in Locally Advanced Gastric Cancer** (NCT06784765) [clinicaltrials.gov](https://clinicaltrials.gov/study/NCT06784765?utm_source=chatgpt.com) | To see whether adding PIPAC preoperatively in patients with locally advanced GC reduces occurrence of peritoneal metastasis. [clinicaltrials.gov](https://clinicaltrials.gov/study/NCT06784765?utm_source=chatgpt.com) | Clinical trial registered, status likely ongoing (no published results yet). [clinicaltrials.gov](https://clinicaltrials.gov/study/NCT06784765?utm_source=chatgpt.com) | Very directly relevant — this is essentially the prophylactic PIPAC in non-metastatic locally advanced gastric cancer that you are interested in. Provides strong rationale for your study. |
| **PIPAC in Multimodal Therapy for Oligometastatic Gastric Cancer** (VER-One, NCT05303714) [centerwatch.com](https://www.centerwatch.com/clinical-trials/listings/NCT05303714/pipac-in-multimodal-therapy-for-patients-with-oligometastatic-peritoneal-gastric-cancer?utm_source=chatgpt.com) | To test PIPAC + systemic chemotherapy vs systemic chemo alone in GC with synchronous positive cytology / low burden of PM (PCI ≤6). Endpoint includes resectability etc. [centerwatch.com](https://www.centerwatch.com/clinical-trials/listings/NCT05303714/pipac-in-multimodal-therapy-for-patients-with-oligometastatic-peritoneal-gastric-cancer?utm_source=chatgpt.com) | Phase III, multicenter, randomized, still recruiting (estimated completion later) [centerwatch.com](https://www.centerwatch.com/clinical-trials/listings/NCT05303714/pipac-in-multimodal-therapy-for-patients-with-oligometastatic-peritoneal-gastric-cancer?utm_source=chatgpt.com) | While this includes patients *with* cytology positivity or low peritoneal metastasis rather than strictly “non-metastatic”, it is close. It may inform on early intervention and what benefit PIPAC might bring in an earlier disease stage. |

**Related but slightly different: Prophylactic HIPEC**

Since PIPAC is newer, many more studies exist for prophylactic HIPEC (heated intraperitoneal chemotherapy) in non-metastatic locally advanced gastric cancer (especially serosa-invasive) to prevent peritoneal relapse. These may serve as precedent / analogues. For example:

- *Role of prophylactic HIPEC in non-metastatic, serosa-invasive gastric cancers* — shows that p-HIPEC reduces peritoneal relapse and improves disease free / overall survival in some studies, but evidence is heterogeneous. [PubMed](https://pubmed.ncbi.nlm.nih.gov/36159214/?utm_source=chatgpt.com)
- Systematic reviews of prophylactic HIPEC in GC. [PubMed+1](https://pubmed.ncbi.nlm.nih.gov/36159214/?utm_source=chatgpt.com)

These HIPEC studies help with justification, showing that intraperitoneal therapies in prophylactic settings *can* have benefit, though with variable quality of evidence.

**Gaps / What is not yet known**

Based on what’s out there, some clear gaps you might cite:

1. **Few (if any) published data** where *PIPAC is used prophylactically* in *non-metastatic gastric cancer with no peritoneal disease* (microscopic or cytology positive) to reduce risk of peritoneal metastasis as a primary endpoint.
2. **Limited data on long-term outcomes** (disease-free survival, overall survival, peritoneal metastasis incidence) in earlier stage disease when PIPAC is used preoperatively or prophylactically.
3. **No clearly randomized controlled trials** published yet in a purely prophylactic context for GC without confirmed peritoneal involvement.
4. **Heterogeneity in patient selection, dosing, timing** (when to apply PIPAC relative to surgery or chemotherapy, what chemo agents, what frequency).
5. **Safety and tolerability in early disease / non-palliative settings** is less documented, although studies like the staging laparoscopy + PIPAC one show encouraging safety.

If you want, I can try to pull up **all the registered or in-progress trials** globally specific to prophylactic PIPAC in non-metastatic GC (with detailed inclusion criteria), so you can benchmark your design. Do you want that?

**You said:**

yes

**ChatGPT said:**

Here are details of **registered / ongoing trials** relevant to *PIPAC in gastric cancer*, especially those close to prophylactic / early-use settings, and some thoughts on how they compare to your design. If helpful, I can also try to compile a table you can put in your manuscript.

**Relevant Registered / Ongoing Trials**

| **Trial / ID** | **Main Objective / Hypothesis** | **Patient Population** | **Key Design Features** | **Status & Relevance to Prophylactic Use** |
| --- | --- | --- | --- | --- |
| **Preventive Use of PIPAC in Locally Advanced Gastric Cancer (NCT06784765)** | To test whether adding PIPAC pre-operatively in locally advanced GC can **reduce incidence of peritoneal metastasis**. [clinicaltrials.gov](https://clinicaltrials.gov/study/NCT06784765?utm_source=chatgpt.com) | Locally advanced gastric cancer (non-metastatic) — patients likely to have risk of peritoneal micrometastasis but not overt peritoneal metastases. [clinicaltrials.gov](https://clinicaltrials.gov/study/NCT06784765?utm_source=chatgpt.com) | PIPAC treatment given before surgery; prophylactic intent. Key endpoints presumably include rate of peritoneal metastasis; design details (randomization, control etc.) to reduce bias. [clinicaltrials.gov](https://clinicaltrials.gov/study/NCT06784765?utm_source=chatgpt.com) | **Very directly relevant**. Likely your strongest benchmark / comparator. If results not yet published, you can cite that it is “ongoing / in planning / recruiting” or whatever its status is. |

| **PIPAC VER-One / NCT05303714** | To evaluate PIPAC + systemic chemo vs systemic chemo alone in patients with gastric cancer and **synchronous positive peritoneal cytology and/or limited peritoneal metastasis** (PCI ≤6). Endpoints include resectability, survival. [centerwatch.com](https://www.centerwatch.com/clinical-trials/listings/NCT05303714/pipac-in-multimodal-therapy-for-patients-with-oligometastatic-peritoneal-gastric-cancer?utm_source=chatgpt.com) | Gastric cancer with minimal peritoneal disease (cytology positive or low PCI), not entirely non-metastatic (some micrometastatic or minimal peritoneal involvement) [centerwatch.com](https://www.centerwatch.com/clinical-trials/listings/NCT05303714/pipac-in-multimodal-therapy-for-patients-with-oligometastatic-peritoneal-gastric-cancer?utm_source=chatgpt.com) | Randomized, phase III, comparing arms with/without PIPAC + systemic chemotherapy; PIPAC given every two systemic chemotherapy cycles; primary endpoint: secondary resectability; secondary: OS, DFS, QoL etc. [centerwatch.com](https://www.centerwatch.com/clinical-trials/listings/NCT05303714/pipac-in-multimodal-therapy-for-patients-with-oligometastatic-peritoneal-gastric-cancer?utm_source=chatgpt.com) | Close to prophylactic intent but not exactly: there is already presence of cytology positivity or minimal detectable peritoneal spread. But this trial is important for design comparison (how they handle minimal disease, randomization, endpoints). |

| **Phase I PIANO trial (PIPAC-OX + Nivolumab) (NCT03172416)** | Safety/tolerability of combining PIPAC-oxaliplatin with systemic immunotherapy (nivolumab) in gastric cancer with peritoneal metastases. [PubMed+2aacrjournals.org+2](https://pubmed.ncbi.nlm.nih.gov/39288528/?utm_source=chatgpt.com) | Gastric cancer with *established peritoneal metastases*, after first-line systemic therapy. [PubMed+1](https://pubmed.ncbi.nlm.nih.gov/39288528/?utm_source=chatgpt.com) | Phase I; meter of dose, side effects, translational endpoints (e.g. immune infiltration). Not prophylactic. [PubMed](https://pubmed.ncbi.nlm.nih.gov/39288528/?utm_source=chatgpt.com) | Less directly relevant but informative on safety/dosing etc. If prophylactic PIPAC is to be used you can cite this for safety in more advanced disease, to argue that earlier stage might be safer / tolerable. |

| **Other trials / registries** | There are other PIPAC trials, registries, and safety dose-finding studies in various GI cancers. For example: the International PIPAC registry NCT03210298 tracks outcomes of PIPAC in multiple cancer types. [clinicaltrials.gov](https://clinicaltrials.gov/study/NCT03210298?utm_source=chatgpt.com) Also the safety of PIPAC paclitaxel (ePIPAC) etc. [clinicaltrials.gov](https://www.clinicaltrials.gov/study/NCT05395910?rank=1&term=PIPAC&viewType=Table&utm_source=chatgpt.com) | These include patients with peritoneal metastasis, recurrent disease, or advanced disease. | Many are non-randomized, single arm, dose escalation / safety; fewer are prophylactic. | Use for context / background; but they do *not* fill the gap of strong data for prophylactic use in non-metastatic gastric cancer. |

**Comparisons & Design Features You Might Borrow / Consider**

From the trials above, here are useful design elements you might extract or compare with your protocol:

- **Inclusion criteria**: The VER-One trial uses PCI ≤6 or positive peritoneal cytology; that is, low disease burden or microscopic spread. If your protocol is truly prophylactic (no cytology positive / no macroscopic disease), that’s even more stringent. But considering cytology positivity could make your trial more feasible, if you want.
- **Randomization & arms**: VER-One randomizes systemic chemo vs systemic chemo + PIPAC. That’s a good comparator; prophylactic trial should consider a control (standard therapy without PIPAC) vs with PIPAC.
- **Endpoints**: In VER-One, primary endpoint is resectability; secondary include survival, disease-free survival, quality of life etc. In prophylactic setting, you’d want incidence of peritoneal metastasis and disease-free survival.
- **Frequency / timing**: PIPAC is administered every two systemic chemo cycles in VER-One; that can help you justify a schedule. Also consider when PIPAC is done relative to systemic chemo and surgery.
- **Safety and dose**: The PIANO trial and other dose-finding/safety trials give data on tolerability of PIPAC in gastric cancer; useful for justifying your agents/doses, safety monitoring etc.

**Key Gaps that Remain (from the Trials Landscape):**

- No published results from *Preventive Use of PIPAC in Locally Advanced Gastric Cancer (NCT06784765)* (as of when I searched) that definitively show that prophylactic PIPAC reduces peritoneal metastasis in non-metastatic GC. If possible, check status (recruiting/completed).
- VER-One includes patients with **some evidence** of minimal peritoneal disease; so the pure prophylactic cohort (no cytology, no peritoneal disease) is less represented in those trials.
- Long-term outcomes (5-year DFS / OS) are rarely available yet in these early or ongoing trials.

If you like, I can compile a **table of all trials in major registries** (ClinicalTrials.gov, EUCTR, WHO) that specifically include “gastric cancer”, “PIPAC”, “no peritoneal metastasis”, “preventive or prophylactic use” so you can show exactly where your study fits. Do you want me to pull that together?

**You said:**

PIPAC offers several advantages such as improved drug distribution throughout the virtual recesses of the abdomen due to aerosolization; deeper tissue penetration and a prolonged local drug exposure; a reduced systemic toxicity thanks to its limited absorption; and the possibility of repeating the procedure, with, additionally, an adequate assessment of the response to the treatment - paraphrase

**ChatGPT said:**

Here are a few clean, manuscript-ready rewrites—pick the tone you like:

**Option A (concise, one sentence):**
PIPAC provides more uniform intraperitoneal drug distribution, deeper tissue penetration with prolonged local exposure, lower systemic toxicity due to limited systemic absorption, and the ability to repeat treatments while enabling robust assessment of therapeutic response.

**Option B (slightly expanded):**
By aerosolizing chemotherapy within the peritoneal cavity, PIPAC achieves more even distribution into anatomic recesses and enhances tissue penetration with sustained local exposure. It also limits systemic absorption—reducing toxicity—and can be safely repeated, allowing consistent evaluation of treatment response.

**Option C (list style):**
PIPAC offers: (i) more uniform distribution across peritoneal recesses, (ii) deeper tissue penetration and prolonged local exposure, (iii) reduced systemic toxicity from limited absorption, and (iv) repeatability with structured assessment of response.
